# Isolation and characterization of trypanosomatids, including *Crithidia mellificae*, in bats from the Atlantic Forest of Rio de Janeiro, Brazil

**DOI:** 10.1371/journal.pntd.0007527

**Published:** 2019-07-10

**Authors:** Diana Azeredo Rangel, Cristiane Varella Lisboa, Roberto Leonan Morim Novaes, Bruno Alves Silva, Renan de França Souza, Ana Maria Jansen, Ricardo Moratelli, André Luiz Rodrigues Roque

**Affiliations:** 1 Laboratório de Biologia de Tripanosomatídeos, Instituto Oswaldo Cruz, Fundação Oswaldo Cruz, Rio de Janeiro/RJ, Brazil; 2 Programa de Pós-Graduação em Biodiversidade e Biologia Evolutiva, Instituto de Biologia, Universidade Federal do Rio de Janeiro, Rio de Janeiro/RJ, Brazil; 3 Fiocruz Mata Atlântica, Fundação Oswaldo Cruz, Rio de Janeiro/RJ, Brazil; 4 Programa de Pós-Graduação em Ecologia e Evolução, Departamento de Ecologia, Universidade do Estado do Rio de Janeiro, Rio de Janeiro/RJ, Brazil; KARI-Trypanosomiasis Res Centre, KENYA

## Abstract

We studied infection by Trypanosomatidae in bats captured in two areas with different degradation levels in the Atlantic Forest of Rio de Janeiro state: Reserva Ecológica de Guapiaçu (REGUA) and Estação Fiocruz Mata Atlântica (EFMA). Furthermore, we evaluated whether the diversity of trypanosomatids changes according to bat diversity and the different levels of preservation in the region. The results showed no influence of the level of preservation on bat species richness (15 and 14 species, respectively), with similar chiropterofauna and higher abundance of two common fruit-eating bat species in the tropics: *Carollia perspicillata* and *Artibeus lituratus*. Of the 181 bat specimens analyzed by LIT/Schneider hemoculture, we detected 24 infected individuals (13%), including one positive *Sturnira lilium* individual that was also positive by fresh blood examination. Molecular characterization using nested PCR targeting the 18 SSU rRNA-encoding gene fragment showed similar trypanosomatid infection rates in bats from the two areas: 15% in REGUA and 11% in EFMA (*p* = 0.46). *Trypanosoma dionisii* was the most frequently detected parasite (54%), followed by *T*. *cruzi* DTUs TcI and TcIV and *Trypanosoma* sp., in Neotropical phyllostomid bats (RNMO63 and RNMO56); mixed infections by *T*. *dionisii*/*T*. *cruzi* TcIII and *T*. *dionisii*/*T*. *cruzi* TcI were also observed. The *T*. *cruzi* DTUs TcI and TcIV are the genotypes currently involved in cases of acute Chagas disease in Brazil, and *T*. *dionisii* was recently found in the heart tissue of an infected child. Surprisingly, we also describe for the first time *Crithidia mellificae*, a putative monoxenous parasite from insects, infecting a vertebrate host in the Americas. Bats from the Atlantic Forest of Rio de Janeiro state harbor a great diversity of trypanosomatids, maintaining trypanosomatid diversity in this sylvatic environment.

## Author summary

Among all mammals carrying trypanosomatids, bats are considered some of the most ancient hosts, and due to their good adaptation to different landscape features, they may play an important role in parasite dispersion. To assess whether the diversity of trypanosomatid species in bats is affected by the level of environmental disturbance, we collected blood from bats in two localities of Rio Janeiro state in Brazil: one with high and another with low human interference (more preserved). The collected blood was subjected to fresh blood tests and hemoculture. Using the 18S RNA gene as a molecular target, we identified 24 bats (13%) infected by trypanosomatids, mostly by *T*. *dionisii*, a trypanosome species commonly found in all Brazilian biomes and highly associated with bats. Three subpopulations (DTUs I, III and IV) of *T*. *cruzi*, the etiological agent of Chagas disease, were also found to infect bats. In addition, we detected for the first time a putative insect-associated parasite, *Crithidia mellificae*, in a mammal host in the Americas. Our work reveals the high diversity of trypanosomatids infecting bats of the Atlantic Forest of Rio de Janeiro state, reinforcing their importance as potential reservoirs of trypanosomatids.

## Introduction

Trypanosomatids (Protozoa: Trypanosomatida: Trypanosomatidae) are parasitic uniflagellated protists that infect plants, invertebrates and vertebrates and have monoxenous or heteroxenous life cycles. The following monoxenous genera are currently recognized: *Herpetomonas*, *Blastocrithidia*, *Crithidia*, *Leptomonas*, *Wallacemonas*, *Sergeia*, *Strigomonas*, *Angomonas*, *Rhynchoidomonas*, *Blechomonas*, *Paratrypanosoma*, *Kentomonas*, *Lotmaria*, *Lafontella*, *Jaenimonas*, *Zelonia*, and *Novymonas* [1]. Heteroxenous genera include *Endotrypanum*, *Phytomonas*, *Leishmania*, and *Trypanosoma* [1]. Monoxenous trypanosomatids are described as being restricted to insects of the orders Diptera, Hymenoptera, Siphonaptera and Hemiptera. However, the monogenetic trait of these protists is not strict because heteroxenous life cycles have been reported for some, as follows: (i) *Leptomonas seymouri* observed in coinfections with *Leishmania donovani* in patients with visceral leishmaniasis [2,3]; (ii) *Leptomonas* sp. and *Herpetomonas samuelpessoai* mixed infection in an HIV-positive patient [4,5]; (iii) members of *Herpetomonas* infecting plants [6]; and (iv) a recent report of *Blastocrithidia* sp. infecting bats [7]. All these findings show that these parasites exhibit an unsuspected adaptability to new environments.

Trypanosomes (*Trypanosoma* spp.) belong to a monophyletic group of heteroxenous parasites that are well adapted to several vertebrate species and are transmitted by blood-sucking vectors, such as leeches, ticks and insects. Due to their public health importance, some of these parasite species have been studied extensively. Among them are the causative agents of Chagas disease and sleeping sickness, *Trypanosoma cruzi* and *Trypanosoma brucei*, respectively. *T*. *cruzi* is found in hundreds of New World mammal species; indeed, this highly heterogeneous parasite displays a complex life cycle involving high host diversity. *T*. *cruzi* genotypes have been grouped into six discrete typing units (DTUs), TcI–TcVI, in addition to a seventh one named Tcbat. All genotypes of *T*. *cruzi* are able to infect humans, and Chagas disease has possible severe clinical outcomes [8]. The geographical distribution of these DTUs varies according to the different scenarios of transmission in which their hosts are involved. *T*. *cruzi* is placed within the *T*. *cruzi* clade, which includes other trypanosome species that infect a diversity of mammals (including humans), bats in particular.

Unlike other mammals, bats (Chiroptera) have morphological and physiological adaptations for powered flight. Although these animals are key contributors to the value of nature to humans [9], bats may be distrusted due to their potential link to human diseases [10]. A trade-off between the evolution of flight and evolution of the immune system and their metabolism may have allowed bats to host many parasites, some of which are deadly to humans [11,12].

Bats from all over the world are parasitized by several species of *Trypanosoma*, with some exclusively infecting these mammals [7,13–17]. Furthermore, recent studies reveal high bat trypanosome diversity [13,17–20], clarifying the ancient origin of the bat-trypanosome interaction. Currently, the most accepted hypothesis explaining the origin of *T*. *cruzi* indicates that this parasite species evolved from an ancestral bat trypanosome [21].

The Atlantic Forest is the second-largest Brazilian biome, encompassing the east coast of the country. Despite severe fragmentation, the Atlantic Forest continues to show high levels of species diversity [22,23]. Although the bat diversity in the Atlantic Forest of Rio de Janeiro has been well studied, very little is known about the diversity of trypanosomatids circulating in bats and the role of bats in maintaining this diversity in the region. Moreover, approximately 70% of Rio de Janeiro’s population lives in the Atlantic Forest area [24], which reinforces the importance of including parasites that can potentially infect humans in studies of local biodiversity.

In this study, we surveyed the diversity of trypanosomatid species that circulate in bats from two areas with different conservation and connectivity levels in the Atlantic Forest of Rio de Janeiro. For the most preserved and larger area (Guapiaçu Ecological Reserve), there has been no indication of trypanosomatid infection. In contrast, the more degraded and smaller area (Fiocruz Atlantic Forest Biological Station) represents an important scenario in terms of the transmission of *T*. *cruzi* among wild mammals and *Leishmania* parasites, with human and canine cases of visceral leishmaniasis having been reported [25]. Our working hypothesis was that the diversity of trypanosomatids varies according to the local chiropterofauna, which is modulated by the level of preservation of the habitat.

## Material and methods

### Ethics statement

Bat samplings were conducted under permits of Instituto Chico Mendes de Conservação da Biodiversidade (19037–1). All collected bats received a number and were deposited in the mammal collection of the National Museum, administered by the Federal University of Rio de Janeiro, Rio de Janeiro, Brazil. Field procedures were approved by the Fiocruz’s Ethics Commission on Animal Use (licenses L-81/12, LW-68/12). All procedures were performed according to biosafety standards, and no environmental damage occurred.

### Study areas and bat sampling

**Fig 1:**
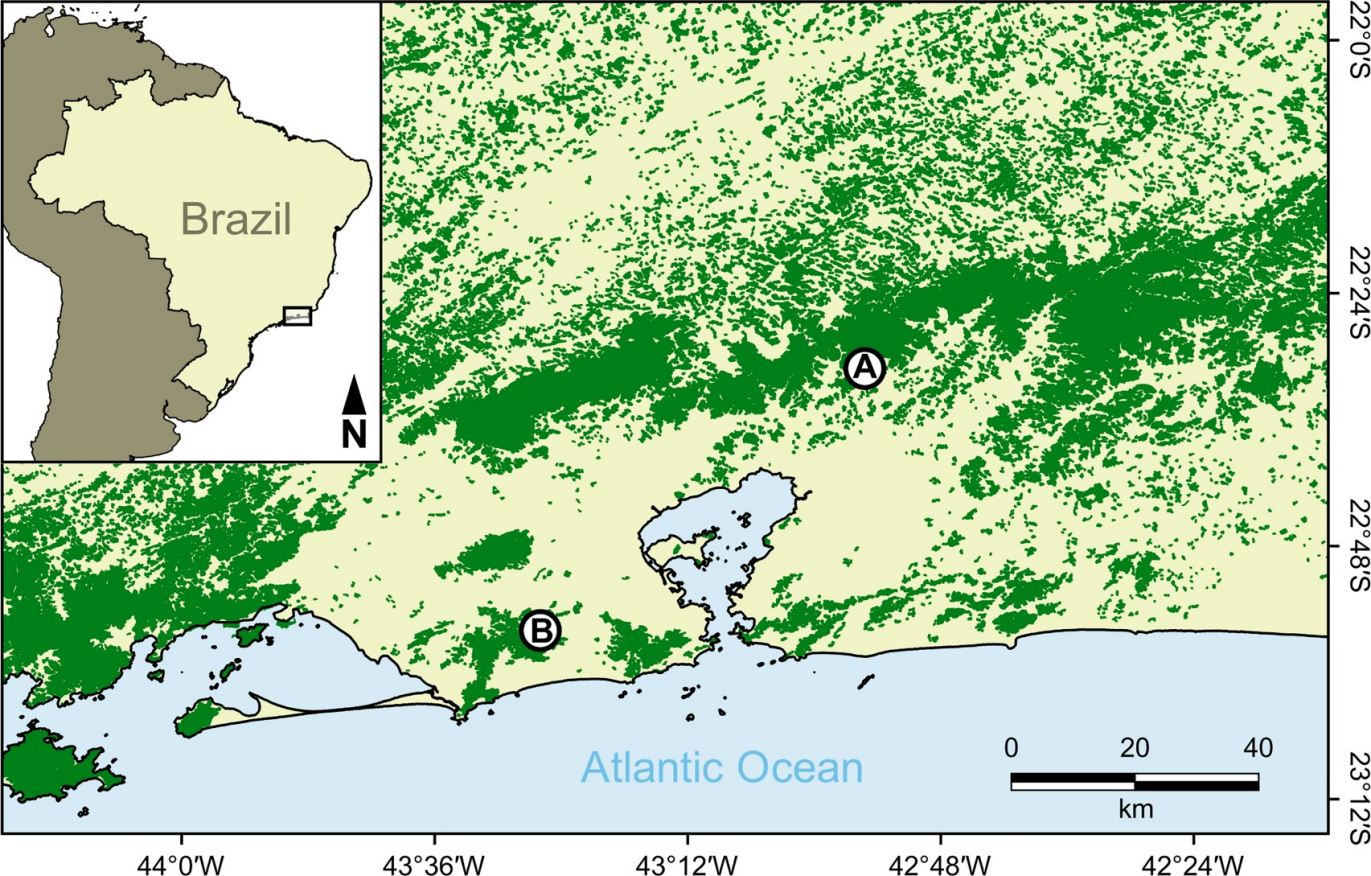
Map of part of Rio de Janeiro state, southeastern Brazil, showing the Atlantic Forest remnants (green) where bats were sampled: Reserva Ecológica de Guapiaçu (A) and Estação Biológica Fiocruz Mata Atlântica (B).

The study was performed in two areas, Reserva Ecológica de Guapiaçu (REGUA [Guapiaçu Ecological Reserve]) and Estação Biológica Fiocruz Mata Atlântica (EFMA [Fiocruz Atlantic Forest Biological Station]), both in the Atlantic Forest of Rio de Janeiro state (Fig 1). REGUA (22°25'53"S, 42°45'20"W) is a 4,000 ha private reserve in Guapiaçu village, Cachoeiras de Macacu municipality. This area is located in the mountainous region of Rio de Janeiro, 110 km away from the Rio de Janeiro municipality, and is widely connected to the largest remnant of Atlantic Forest in the state, with a total of more than 20,000 ha of continuous forests surrounded by matrixes of pastures and plantations. EFMA (22º56'23"S, 43º24'12"W) is located within the core of the Rio de Janeiro municipality, comprising 480 ha in the Pedra Branca Massif (~ 6,000 ha). The Pedra Branca Massif is fully isolated from other fragments and surrounded by low-income communities.

Three sampling sites representing a forest structure gradient from the border to the interior of the forest were established in each area. These sites were defined as follows: (P1) forest edge with initial secondary forest and high anthropogenic intervention, (P2) initial secondary forest with low anthropogenic intervention, and (P3) late secondary forest with low anthropogenic intervention.

### Bat and trypanosomatid samplings

From September to December 2015 and February to May 2016, bats were sampled every two months in each area, covering both dry and wet seasons. Ten mist nets (Zootech 9×3 m, 20 mm mesh) were used per night, with one night of sampling at each sampling site (totaling three nights per area every two months). Ground-level mist nets were placed along trails, in forest clearings and on watercourses, remaining open for five hours after sunset. Eight sampling campaigns were carried out, totaling 12 sampling nights for each area of capture. Bats were removed from the nets, sexed and measured [26]. Some bats were released after being marked with numbered bands on the forearm, and some were collected for the trypanosomatid survey according to the capture license. These bats were kept in individual cotton bags and processed the next day in the field laboratory.

In the following morning, the bats that were selected for the trypanosomatid survey were anesthetized using a combination of 10% ketamine hydrochloride and 2% acepromazine (9:1). After this procedure, the body was sanitized using bactericidal soap, iodized alcohol and 70% alcohol. Approximately 0.5–1 mL of blood was collected using heart puncture for the following procedures: (i) inoculation of one tube containing NNN/LIT (Novy-MacNeal-Nicolle/liver infusion tryptose) medium and another tube containing NNN/Schneider medium (0.2–0.4 mL in each tube) and (ii) a fresh blood examination. The anesthetized bats were euthanized after blood collection using direct application of potassium chloride to the heart. The collected specimens were deposited in the Museu Nacional, Universidade Federal do Rio de Janeiro. Bat identification followed Gardner [27], and nomenclature followed Nogueira et al. [28].

### Parasitological procedures and DNA extraction

Fresh blood examination was performed in the field based on visual observation of a drop of blood on microscope slides using optical microscopy (400×). The samples that presented flagellates with a morphology similar to that of a *Trypanosoma* sp. were considered positive.

Hemocultures performed in the field were incubated at 28ºC for 10 days, after which the cultures were examined using an optical microscope every 14 days for up to 5 months. The positive blood cultures from which isolate flagellate parasites were identified were amplified and cryopreserved in the Collection of *Trypanosoma* from Wild and Domestic Mammals and Vectors (COLTRYP) (www.coltryp.fiocruz.br). Some positive blood cultures in which parasites were observed but for which cultures were not established were transferred to another tube (1.5 mL), centrifuged for 15 min, and stored at -20ºC for molecular characterization.

DNA extraction from positive hemocultures was performed using the phenol/chloroform method according to Sambrook and Russell [29]. At the end of this procedure, 150 μL or 40 μL of Tris-EDTA buffer solution (pH 8.0) was added to the DNA extracted from the hemocultures from which parasites were isolated or were not isolated, respectively.

Culture smears of the LBT7439 sample were stained with Giemsa and observed under a Zeiss Axioplan microscope (Oberkochen, Germany).

### Molecular characterization and phylogenetic analyses

Parasite identification was performed using 18 SSU nested PCR according to Noyes et al [30]. In the first step, we used GoTaq Hot Start Green Master Mix (Promega), followed by the addition of 2 μL of DNA to 0.5 μL of each primer (Oligo Try R and Oligo Try F; TRY927F 5’GAAACAAGAAACACGGGAG3’ and TRY927R 5’CTACTGGGCAGCTTGGA3’) at 16 pmol, 12.5 μL of GoTaq Master Mix and ultra-pure water for a total volume of 25 μL. The reaction was performed under the following conditions: initial denaturation at 95ºC for 15 min and 30 cycles of 94ºC for 30 s, 55ºC for 60 s and 72ºC for 92 s. The expected fragment size for this step was 900 bp. In the second step, we used primers at the same concentrations (SSU561F 5’TGGGATAACAAAGGAGCA3’ and SSU561R 5’CTGAGACTGTAACCTCAAAGC3’). The product from the first step (2 μL) was used as the template for the second step. To obtain better results, we used 5 μL for all reactions for DNA samples derived from hemocultures from which no parasites were isolated. The expected fragment size for this step was 600 bp. Amplification using these primers did not occur for two DNA samples, and we thus tested the gGAPDH gene as the target according to Borghesan et al. [6]. To each 2 μL of sample DNA, 1 μL of each primer (0.4 μM; GAPTRY-F 5’GGBCGCATGGTSTTCCAG’3 and GAPTRYr-R 5’CCCCACTCGTTRTCRTACC’3) was added, in addition to 2.5 μL of buffer, 0.75 of MgCl_2_, 2 μL of dNTPs, 0.3 Taq Platinum and ultra-pure water for a total volume of 25 μL. The reaction was performed under the following conditions: initial denaturation at 94ºC for 3 min and 30 cycles of 94ºC for 1 min, 55ºC for 2 min and 72ºC for 2 min. The expected fragment size was approximately 800 bp.

The amplified PCR products were visualized using ethidium bromide-stained agarose gel electrophoresis (2%). Purification was performed for positive samples using illustra GFX PCR DNA/Gel Band Purification Kit (GE, Healthcare). DNA sequencing was performed with ABI 3730 BigDye Terminator v3.1 Cycle Sequencing Ready Reaction Kit (Applied Biosystems, California, USA) using internal primers at 3.2 pmol and an Applied Biosystems DNA Analyzer at the Oswaldo Cruz Foundation Sequencing Platform (PDTIS/FIOCRUZ).

The obtained sequences were manually edited using the SeqMan (DNAstar) [31] program. To identify species, we used sequences with both coverage and identity equal to or greater than 98%. Sequence alignment was performed using BioEdit [32] software. For phylogenetic analysis, we applied the Kimura 2-parameters model (K2 + G) (Kimura, 1980) [33] for the 18S target and neighbor-joining (NJ) method (Saitou and Nei, 1987) [34] and the Tamura Nei model (TN 9 + G) (Tamura and Nei, 1993) [35] for the gGAPDH target, both using jModel Test program 2.1. The probabilistic method of maximum likelihood (ML) was employed for both 18S and gGAPDH. Only those sequences that matched equal or greater than 98% identity without double peaks in the electropherogram were included in the ensuing phylogenetic analyses.

### Diversity indexes and statistical analysis

We utilized two diversity indexes to assess differences in species evenness between the sampling points (Shannon H and Simpson D), one to examine differences in richness (Margalef) and two to verify differences in dominance and equitability. We performed Kruskal-Wallis one-way analysis to evaluate distribution for the samples, whereby significance (p < 0.05) indicates that at least one sample stochastically dominated one other sample. To assess the association between the level of preservation and bat infection, we performed chi-square tests on contingency tables cross-classifying infection (infected/not infected) by the sampling points in each area; results with a *p*-value smaller than 0.05 were considered significant.

## Results

A total of 181 bats (113 males and 68 females) of 18 species were surveyed for trypanosomatid infection in both areas. Among them, 116 bats (64%) were captured in REGUA and 65 (36%) in EFMA. Most species of bats included representatives of Phyllostomidae (17 species), in contrast to the Vespertilionidae (one species, *Myotis riparius*) family (Table 1). *Carollia perspicillata* and *Artibeus lituratus* were the most frequent species in both areas and represented the largest samples of specimens examined for trypanosomatids. Diversity indices indicated lower evenness and richness for most preserved sampling sites (P3). Despite differences in richness and diversity, Kruskal-Wallis tests did not show significant differences between the sampling areas (*p* = 0.51) or among the sampling sites within EFMA (*p* = 0.54) and REGUA (*p* = 0.24).

**Table 1:** Bat species examined at each area (Estação Biológica Fiocruz Mata Atlântica [EFMA] and Reserva Ecológica Guapiaçu [REGUA]) and sampling site (P1, P2 or P3).

	Area/sampling sites						Trophic guild (Kalko 1996)[36]
Bat species	EFMA			REGUA			
	P1	P2	P3	P1	P2	P3	
*Anoura caudifer*	–	–	1	2	1	–	Nectarivorous
*Anoura geoffroyi*	–	–	–	2	–	–	Nectarivorous
*Artibeus fimbriatus*	–	–	1	1	–	–	Frugivorous
*Artibeus lituratus*	8	2	6	7	8	3	Frugivorous
*Artibeus obscurus*	1	1	–	1	1	–	Frugivorous
*Carollia perspicillata*	9	11	9	11	26	17	Frugivorous
*Chrotopterus auritus*	–	–	–	1	–	–	Animalivorous
*Desmodus rotundus*	1	2	1	1	4	1	Hematophagous
*Glossophaga soricina*	–	1	–	–	–	–	Nectarivorous
*Lonchophylla peracchii*	–	1	–	–	–	–	Nectarivorous
*Lonchorrina aurita*	–	–	–	–	1	–	Insectivorous
*Micronycteris microtis*	–	1	–	–	–	–	Insectivorous
*Myotis riparius*	–	–	–	1	–	–	Insectivorous
*Phyllostomus hastatus*	2	–	–	5	–	–	Omnivorous
*Platyrrhinus recifinus*	-	–	1	–	–	1	Frugivorous
*Sturnira lilium*	1	3	–	8	6	4	Frugivorous
*Sturnira tildae*	1	–	–	–	–	2	Frugivorous
*Vampyressa pusilla*	–	1	–		–	1	–		Frugivorous
Total number of individuals	23	23	19	40	48	28	
Total number of species	7	9	6	11	8	6	

Only one blood sample from an *S*. *lilium* individual (LBT7559) captured in REGUA presented flagellate parasites upon fresh blood examination. Infection of this bat was also confirmed by parasite isolation using both LIT and Schneider culture media, and the flagellates were characterized as *Trypanosoma dionisii*.

**Fig 2:**
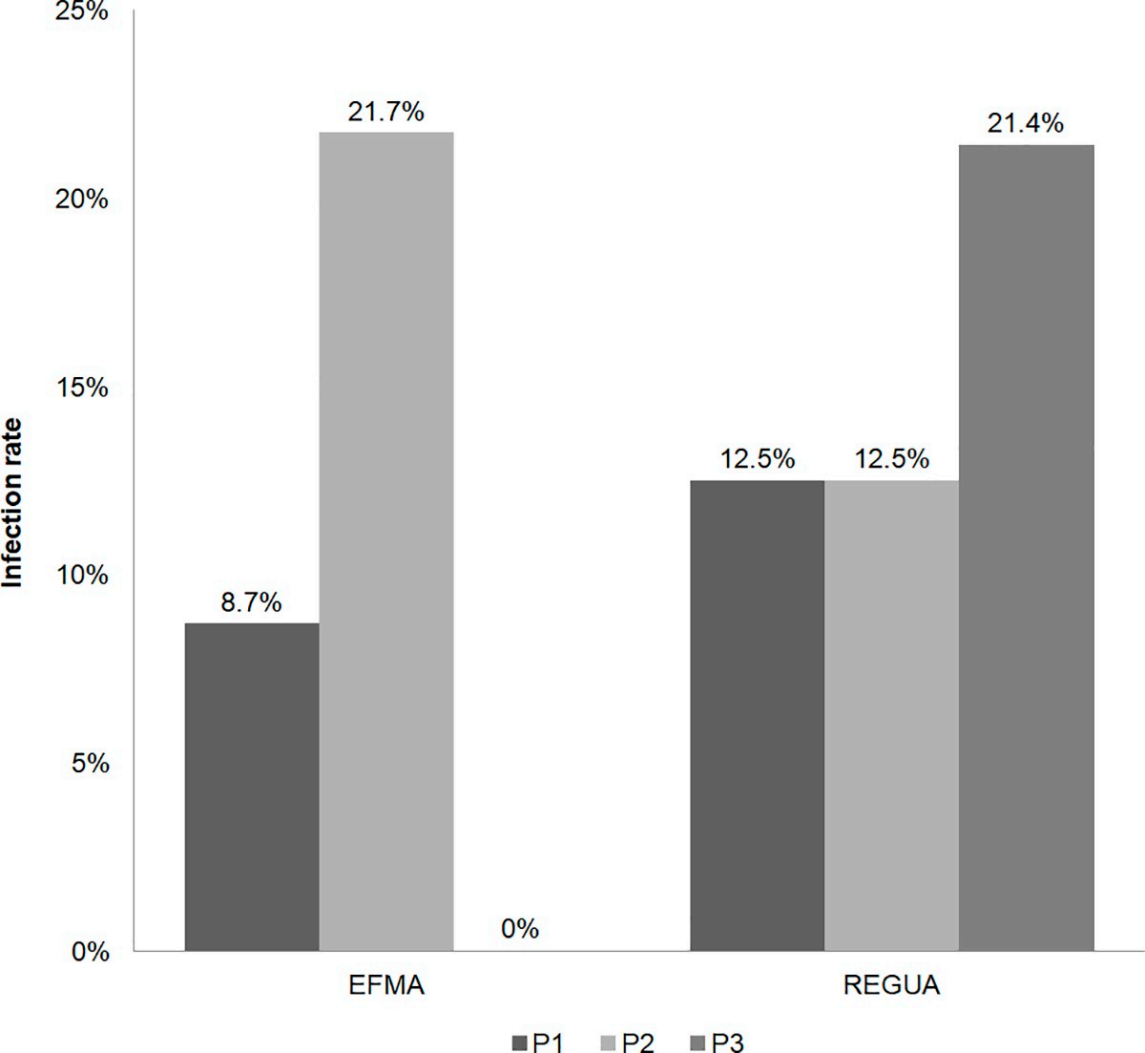
Trypanosomatid infection rate in bats captured at REGUA (Reserva Ecológica Guapiaçú) and EFMA (Estação Biológica Fiocruz Mata Atlântica), Rio de Janeiro state, Brazil. Infection rates by sampling site for each study area are shown in columns. Sites P1, P2 and P3 are represented by colors.

Overall, 24 bats (13%) were positive for trypanosomatids, with no significant difference between the studied areas: 15% (n = 17) and 11% (n = 7) for REGUA and EFMA, respectively (*p* = 0.46). Moreover, significant differences in infection rates among the sampling sites within each area were not observed (*p* = 0.5 for REGUA and *p* = 0.07 for EFMA) (Fig 2). Trypanosomatid infection was detected in 6 bat species: *Carollia perspicillata*, *Artibeus lituratus*, *Sturnira lilium*, *Desmodus rotundus*, *Anoura caudifer* and *A*. *geoffroyi*. Flagellate parasites were detected in LIT (42%, n = 10 bats), Schneider (17%, n = 4 bats) or both types (42%, n = 10 bats) of culture media. Parasites were isolated from 17 blood cultures and were cryopreserved and deposited in COLTRYP. Another 16 positive cultures did not yield in cryopreserved isolates but were molecularly analyzed by PCR using a single culture (LIT medium from LBT7663). *T*. *cruzi-*like flagellates were observed by light microscopy, but it was not possible to isolate flagellates to obtain sufficient DNA for molecular analysis (Table 2).

**Table 2:** Trypanosomatid species infecting bats. This table shows the bat identification number, area (Reserva Biológica de Guapiaçu/REGUA or Estação Biológica Fiocruz da Mata Atlântica/EFMA), bat species, positive culture media, culture media in which the parasite was isolated and cryopreserved at COLTRYP (with their respective numbers) and the characterized parasite species and GenBank accession number.

LBT	Area	Bat species	Media*	Isolate*	COLTRYP number	18S SSU gene characterization	GenBank accession number
7439	REGUA	*Anoura caudifer*	L	L	685	Crithidia mellificae^**^	MH469963
7149	REGUA	*Anoura geoffroyi*	L/S	-	-	*T*. *dionisii* (L/S)	MH469960/MH469961
6873	REGUA	*Carollia perspicillata*	L	-	-	*Trypanosoma* sp.^1^**	*-*
6890	REGUA	*Carollia perspicillata*	L	-	-	*Trypanosoma* sp^.^^2^	MH469953
7578	REGUA	*Carollia perspicillata*	L	-	-	*Trypanosoma* sp.^1^	*-*
6882	REGUA	*Carollia perspicillata*	L	L	642	*T*. *cruzi* TcI	MH469942
7557	REGUA	*Carollia perspicillata*	L	L	706	*T*. *dionisii*	MH469949
7137	REGUA	*Carollia perspicillata*	S	-	-	*T*. *dionisii*	MH469958
7455	REGUA	*Carollia perspicillata*	S	S	698	*T*. *dionisii*	MH469948
6889	REGUA	*Desmodus rotundus*	L	L	639	*T*. *dionisii*	MH469940
7118	REGUA	*Desmodus rotundus*	L/S	-	-	*Trypanosoma* sp^.^^2^ (L/S)	MH469955/MH469956
7123	REGUA	*Desmodus rotundus*	S	-	-	*Trypanosoma* sp.^2^	MH469957
6875	REGUA	*Sturnira lilium*	L	L	638	*T*. *dionisii*	MH469939
7138	REGUA	*Sturnira lilium*	L/S	-	-	*Trypanosoma* sp.^1^ (L/S)	*-*
7148	REGUA	*Sturnira lilium*	L/S	L	693	*T*. *dionisii* (L)*/T*. *cruzi* TcI(S)	MH469947/MH469959
7559	REGUA	*Sturnira lilium*	L/S	L/S	708	*T*. *dionisii* (L/S)	MH469951/MH469952
7128	REGUA	*Sturnira lilium*	S	-	-	*T*. *cruzi*	*-*
6967	EFMA	*Artibeus lituratus*	L/S	L	645	*T*. *cruzi* TcIII (L)*/T*. *dionisii* (S)	MH469946/MH469954
7668	EFMA	*Carollia perspicillata*	L	-	-	*T*. *cruzi* TcIV	MH469962
7666	EFMA	*Carollia perspicillata*	L	L	709	*Trypanosoma* sp.^*1*^	*-*
6968	EFMA	*Carollia perspicillata*	L/S	L	641	*T*. *dionisii* (L/S)	MH469941
7663	EFMA	*Carollia perspicillata*	L/S	S	707	*T*. *dionisii* (S)	MH469950
6973	EFMA	*Desmodus rotundus*	L/S	L/S	644	*T*. *dionisii* (L/S)	MH469944/MH469945
6969	EFMA	*Sturnira lilium*	L/S	L/S	643	*T*. *dionisii* (S)/T*rypanosoma* sp.^*1*^(L)	MH469943

* L = LIT; S = Schneider

** target gene—gGAPDH

^1^ *Trypanosoma* sp. not included in the phylogenetic tree due to the low identity observed by Blast search (<98%) or the presence of double peaks in sequence analysis.

^2^ *Trypanosoma* sp. included in the phylogenetic tree.

*T*. *dionisii* was found in the majority of the infected bats (54%; n = 13), which included *C*. *perspicillata* (n = 5), *S*. *lilium* (4), *D*. *rotundus* (2), *A*. *lituratus* (1) and *A*. *geoffroyi* (1), with eight from REGUA and five from EFMA. One individual of *Sturnira lilium* (LBT7148) and one of *A*. *lituratus* (LBT6967) presented mixed infections by *T*. *cruzi* DTUs I and III, respectively (Table 2).

**Fig 3:**
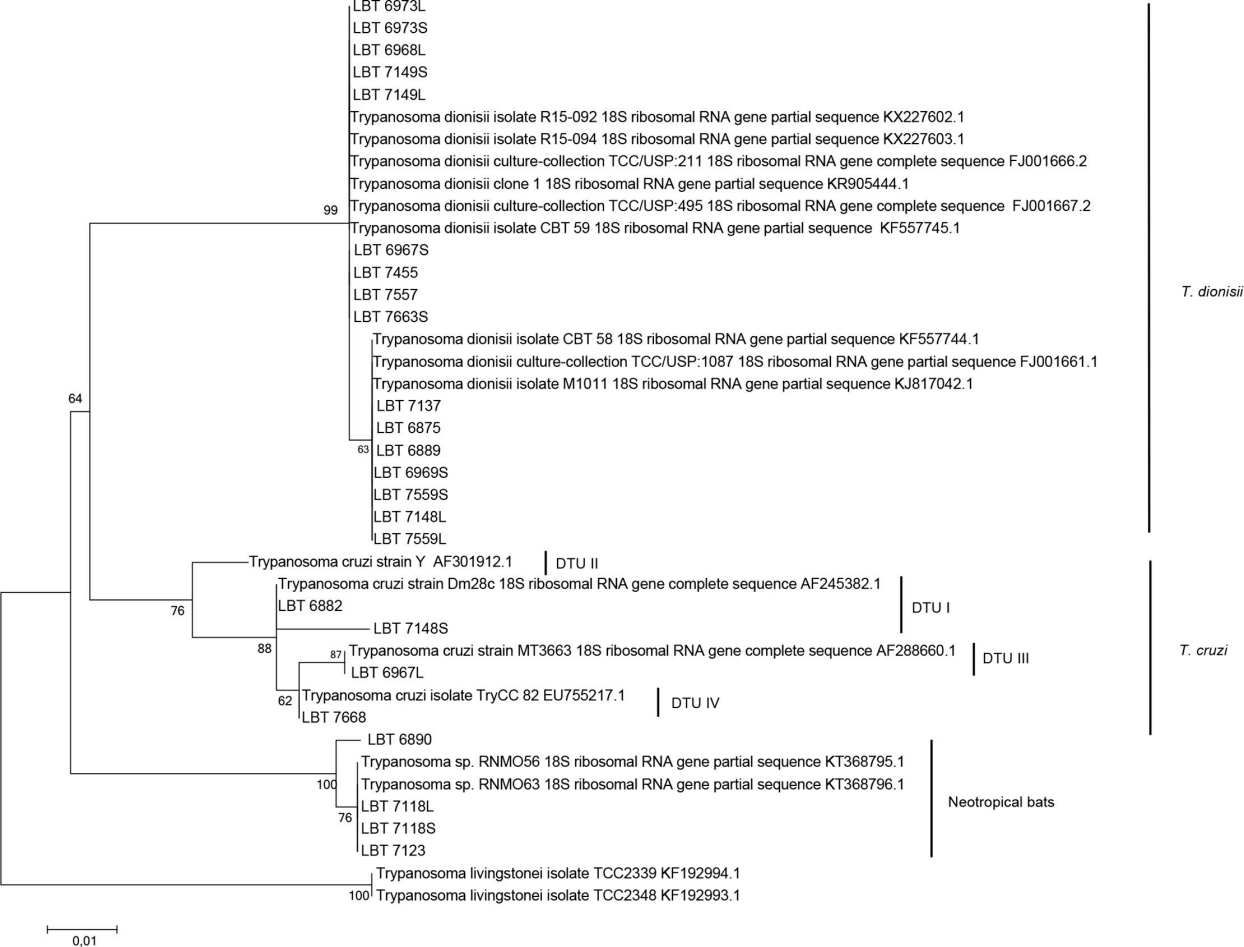
Molecular phylogenetic analysis of the parasite species detected in bats using maximum likelihood based on the 18S SSU rRNA gene with the K2 + G parameters and a bootstrap value of 1000 replicates. *T*. *livingstonei* isolates TCC 2339 and TCC 2348 were used as outgroups.

Four samples of *C*. *perspicillata* and *D*. *rotundus* (LBT6890, LBT7118 and LBT7123) were infected with Neobat trypanosomes (Fig 3). Identity to sequences from species belonging to *Trypanosoma* was found for another five samples of *C*. *perspicillata* (n = 3) (LBT7578, LBT7666, and LBT6873) and *S*. *lilium* (n = 2) (LBT7138 and LBT6969); however, it was not possible to identify the species.

**Fig 4:**
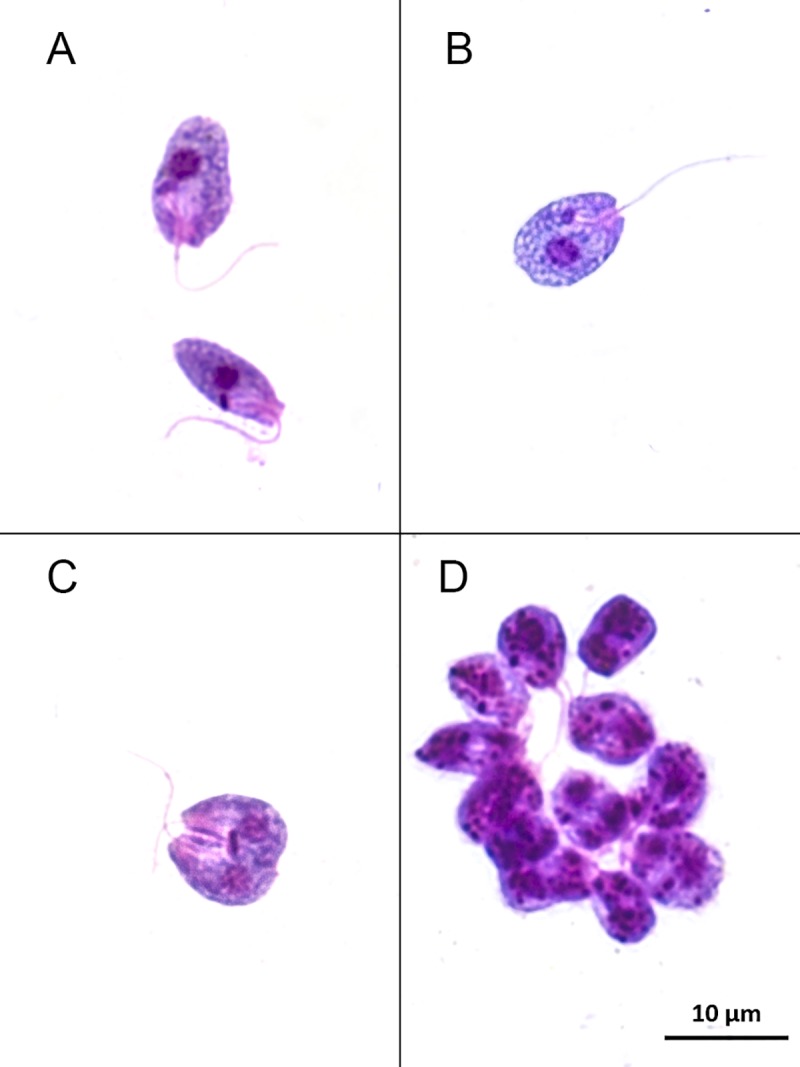
Choanomastigote forms observed in axenic culture of the LBT 7439 sample. (A) Parasite cells show a typical collar-like extension through which a single flagellum emerges. (B) The kinetoplast is anterior to the nucleus and adjacent to the flagellar pocket where emergence of the flagellum can be observed. (C) Dividing forms. (D) Rosette of flagella.

**Fig 5:**
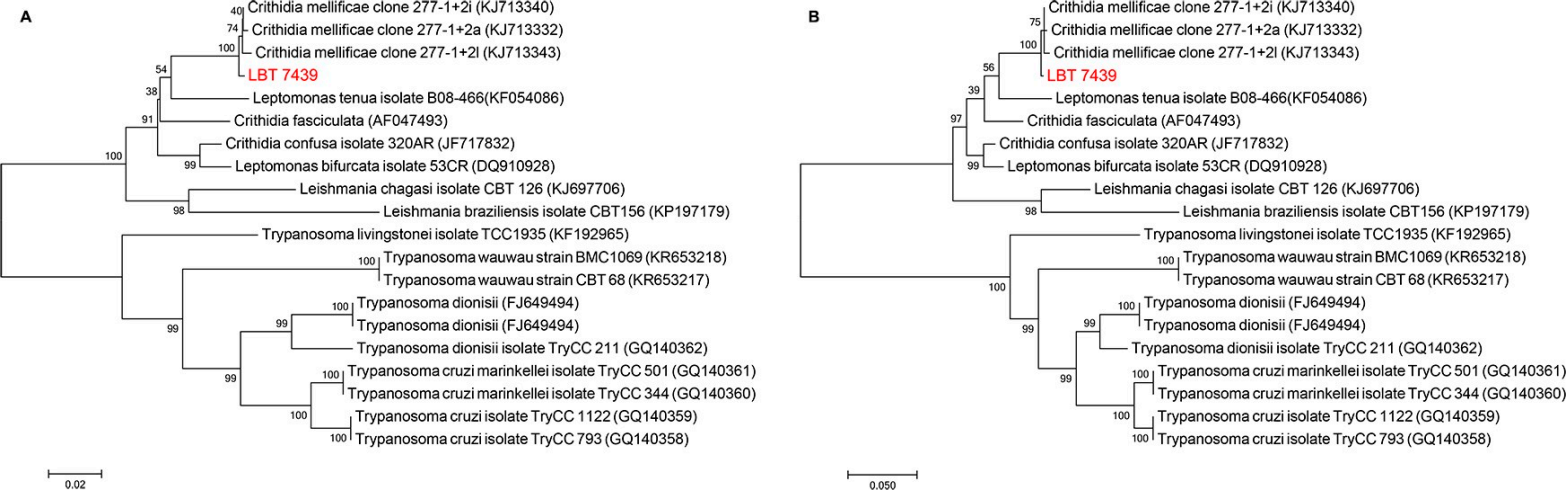
Molecular phylogenetic analysis of the LBT 7439 sample. (A) Analysis using neighbor-joining of the gGAPDH gene with the K2 parameter and a bootstrap value of 1000 replicates. (B) Analysis of the gGAPDH gene using maximum likelihood with Tamura-Nei (93 + G) and a bootstrap value of 1000 replicates.

The parasites isolated from one individual of *Anoura caudifer* (LBT7439) from REGUA presented a peculiar choanomastigote morphology (Fig 4). Sequencing analysis of the products derived from 18S SSU gene PCR suggested a monoxenous parasite infection, but with low identity. The product revealed 88% identity with deposited *Crithidia mellificae* strains and with other putative insect parasites, such as *C*. *expoeki*, *C*. *bombi* and *Leptomonas* spp. Identification of this isolate as being the putative monoxenous trypanosomatid species *C*. *mellificae* was achieved with 99% identity using gGAPDH PCR (Table 2; Figs 3 and 5; S1 Text; S2 Text).

## Discussion

Bats have been described as hosts and/or potential reservoirs of trypanosomes and other microorganisms, such as viruses and bacteria [10,37,38]. Here, we compare trypanosomatid infection in bats from two areas with different levels of preservation and connectivity in the Atlantic Forest of Rio de Janeiro state.

Detection of parasites in one fresh blood examination (LBT 7559) and in blood cultures (LIT/Schneider) revealed that bats may maintain, at least for a time period, detectable parasitemia by the *Trypanosoma* species isolated in this study. Thus, bats may act as an infection source of these parasite species for vectors, as no more than 0.3 mL of blood was cultured and only one drop was used for the fresh blood examination. This also indicates the important role played by infected bats in the maintenance in nature of the trypanosomatid species detected [39]. We observed a sylvatic transmission cycle of distinct trypanosomatid species occurring among bats in REGUA and EFMA. Bats from the most preserved larger area (REGUA) showed slightly higher infection rates than did those from the most degraded and smaller areas (EFMA), 15% and 11%, respectively. The relationship between hemoparasite infection rates and bat species richness in response to fragmented or disturbed habitats was previously studied by Cottontail et al. [40] and Costa et al. [41], who reported a negative correlation between species richness and infection rate, supporting the Dilution Effect proposed by Keesing [42]. Thus, we would expect lower infection rates with higher bat species richness.

The shelters used by bats may reveal their parasite transmission dynamics. Twenty-three of 24 infected bats use a wide variety of shelters, including hollow trees, caves, palm leaves and artificial constructions. Only *A*. *lituratus* uses palm trees as shelter [43]. As Triatominae may also utilize these shelters, such behavior may increase the chances of occasional encounters between bats and *T*. *cruzi* vectors. A different scenario occurs in the Amazon biome, where the most important *T*. *cruzi* vectors are *Rhodnius* spp. [44–46]. The infection rate among bats roosting in palm trees appears to be higher in the Amazon compared to the Atlantic Forest [14,41,47].

The most frequent species in the study areas (*C*. *perspicillata*, *S*. *lilium* and *D*. *rotundus* [48,49]) presented the highest infection rates. These bats are well adapted to different landscapes. In South America, *C*. *perspicillata* has been reported as the species most infected by *T*. *cruzi* DTUs I, III, IV and V, as well as by *T*. *c*. *marinkellei*, *T*. *dionisii*, and *T*. *rangeli* [14,15,18,41,50–54]. In Brazil, *S*. *lilium* has been found to be infected by *T*. *dionisii* and *T*. *cruzi* [55,56]. In this sense, four distinct *T*. *cruzi* lineages able to infect humans were already associated with the most frequent bat species captured in the study areas. Nonetheless, these findings only reflect the high abundance of these bat species in the tropics, not necessarily the association between them and those parasites.

It is impossible to evaluate the impact of trypanosomatid infection on bat fitness and survival rates. Moreover, it is worth considering that all wild mammalian surveys include only those animals that preserved their ability to dislocate, which involves a unavoidable bias [54,57]. As expected, *T*. *dionisii*, described as infecting bats in both the New and Old Worlds, was the trypanosomatid species most detected, and the ability to infect a high diversity of bats, including at least 20 species representing five families and different trophic guilds, has been described for *T*. *dionisii* [20,53,54]. In Brazil, *T*. *dionisii* has been found to infect the phyllostomids *C*. *perspicillata* and *S*. *lilium* as well as noctilionids, molossids and vespertiolionids [54], though the means of transmission remains unclear. *T*. *dionisii* has also been reported to be a unique vector for the Old World parasite *Cimex pipistrellus* [58]. Conversely, bed bugs serve as vectors for another *Trypanosoma* species, *T*. *vespertilionis*, which is restricted to the Old World. Recently, our group detected *T*. *dionisii* infection in triatomines (*Triatoma vitticeps*) from the Guarapari municipality, Espírito Santo, Brazil [15]. This finding shows that *T*. *cruzi* and *T*. *dionisii* might share vectors in the New World.

We detected mixed infection by *T*. *dionisii* and *T*. *cruzi* TcI and TcIII in *S*. *lilium* and *A*. *lituratus*, respectively. Detection from blood samples reveals the capacity of the host to present high parasitemia, being a competent reservoir to transmit those parasites to a vector. We also detected single infections by *T*. *cruzi* DTUs TcI and TcIV in *C*. *perspicillata*. The DTUs TcI, TcIII and TcIV are often found in bats [54,59], even in mixed infections, such as TcI/TcIV, which is the second most common association in nature. The DTU TcI is considered to be the most widespread and frequent DTU in nature [60]. In recent acute cases of Chagas disease in Brazil (Chagas disease outbreaks), human isolates were genotyped as TcI and TcIV [61].

We also successfully isolated *C*. *mellificae* for the first time in South America. *C*. *mellificae* was first described as infecting bees (*Apis mellifera*) in Australia in 1967 and for many years was considered to be the main agent of honey bee colony losses around the world [62,63]. This monoxenic species appears to be able to infect a large diversity of representatives of the Hymenoptera order because it has been found in *Vespula squamosa* (wasp; Vespidae), *Osmia cornuta* (orchard bee; Megachilidae) and *O*. *bicornis* (mason bee; Megachilidae) in the United States [64].

We speculate two possible scenarios for explaining infection of *Anoura caudifer*, a primarily nectar-feeding bat [65]: (i) the bat may have become infected through ingestion of pollen contaminated with bee excreta, as this parasite is found in the posterior portion of the bee’s digestive tract; or (ii) an infected bee may have shed its stinger, which normally includes the posterior portion of its digestive tract, in the bat, and it then became infected by licking the sting site. Moreover, there are two unexpected and original aspects to consider: one is the first description of this monoxenic trypanosomatid in South America, and the other is the first isolation of a *Crithidia* species from a mammalian host. This highlights the necessity of (1) carefully reevaluating the current geographical distribution of *C*. *mellificae*, (2) attempting to understand whether this parasite is expanding its worldwide distribution or if it merely has never been previously noticed and (3) reevaluating the host specificity of trypanosomatids and their competence for spill over for other taxa. Regardless, the leap that constitutes adapting from the digestive tube of an insect to the circulatory system of a mammal is dramatic.

For both proposed scenarios, the infection would have occurred through the oral route, despite the rare opportunity for these animals to interact in nature because they have different foraging behaviors in terms of the timing of activity. A recent study conducted in Texas, USA, detected for the first time another monoxenous trypanosomatid, *Blastocrithidia* sp., infecting bats [7]. The authors described this parasite in the cardiac tissues of the insectivorous bats *Tadarida brasiliensis* (Molossidae) and *Nycticeius humeralis* (Vespertilionidae). Although not isolated, detection of this monoxenous parasite in cardiac tissue, along with our results, reveal the potential of these monoxenous parasites to successfully infect mammalian hosts.

Apart from the surprising isolation of *C*. *mellificae*, we still have scant information about the real diversity of trypanosomatid species that are able to infect bats. The pattern of infection by a parasite may change among its hosts, which is the reason why host biology should be important in parasitological studies. Considering the high diversity of trypanosomatid species and/or genotypes isolated from blood cultures, we conclude that bats are extremely resilient to infection by trypanosomatids and can be sources of infection to vectors, being able to maintain a high diversity of these parasites.

Our study provides an overview of trypanosomatid diversity in bats from two areas with different levels of human disturbance. In both areas, the most frequent species, *Carollia perspicillata* and *Sturnira lilium*, serve as reservoirs of *T*. *cruzi* and *T*. *dionisii*, both of which are able to infect humans. Moreover, the most frequently detected *T*. *cruzi* DTUs in bats (TcI and TcV) are most commonly associated with new Chagas disease cases in Brazil [61]. The finding of a putative monoxenous parasite (*C*. *mellificae*) infecting bats reinforces the urgency of discussing the concepts of parasitism and hosts among trypanosomatids. These bat species that were more frequent in the two areas exhibit plasticity in habitat usage, occurring in forests with different levels of preservation as well as in urban areas. Our data show that they are also flexible in their capacity to maintain a high diversity of trypanosomatid parasites.

## Supporting information

S1 Text gAPDH aligment. Aligment of gGAPDH GenBank reference sequences used in *Crithidia mellificae* phylogenetic trees.
(TXT)

S2 Text 18 SSU aligment. Aligment of 18S rRNA GenBank reference sequences used in *Trypanosoma* spp. phylogenetic trees.
(TXT)
